# A Neural Network Approach for Building An Obstacle Detection Model by Fusion of Proximity Sensors Data

**DOI:** 10.3390/s18030683

**Published:** 2018-02-25

**Authors:** Gonzalo Farias, Ernesto Fabregas, Emmanuel Peralta, Héctor Vargas, Gabriel Hermosilla, Gonzalo Garcia, Sebastián Dormido

**Affiliations:** 1 Pontificia Universidad Católica de Valparaíso, Avenida Brasil 2147, Valparaíso 2362804, Chile; emmanuel.peraltah@gmail.com (E.P.); hector.vargas@pucv.cl (H.V.); gabriel.hermosilla@pucv.cl (G.H.); 2 Departamento de Informática y Automática, Universidad Nacional de Educación a Distancia, Juan del Rosal 16, 28040 Madrid, Spain; efabregas@bec.uned.es (E.F.); sdormido@dia.uned.es (S.D.); 3 Radar Research and Innovations, 11702 W 132nd Terrace, Overland Park, KS 66213, USA; garciagarreton@hotmail.com

**Keywords:** proximity sensors, automatic calibration, neural networks

## Abstract

Proximity sensors are broadly used in mobile robots for obstacle detection. The traditional calibration process of this kind of sensor could be a time-consuming task because it is usually done by identification in a manual and repetitive way. The resulting obstacles detection models are usually nonlinear functions that can be different for each proximity sensor attached to the robot. In addition, the model is highly dependent on the type of sensor (e.g., ultrasonic or infrared), on changes in light intensity, and on the properties of the obstacle such as shape, colour, and surface texture, among others. That is why in some situations it could be useful to gather all the measurements provided by different kinds of sensor in order to build a unique model that estimates the distances to the obstacles around the robot. This paper presents a novel approach to get an obstacles detection model based on the fusion of sensors data and automatic calibration by using artificial neural networks.

## 1. Introduction

Obstacle avoidance is one of the main challenges for any practical design in robotics. Many approaches to face this problem can be found in the literature. Some of them use optical sensors and computer vision for general purpose object detection tasks [1,2,3]. In particular, proximity sensors are a widely-implemented solution for obstacle detection and collision avoidance in mobile robotics. These sensors are normally located around the robot in order to detect and avoid objects when it navigates in a dynamic environment [4,5,6].

The proximity sensors are able to detect the presence of nearby objects without physical contact, providing an estimation of the distance to the obstacles. Commonly, this kind of sensor emits electromagnetic fields (e.g., infrared light) or sound pulses (e.g., ultrasonic), in order to detect changes in the field or returned signal [7]. For this reason, the surface properties of the objects have an important influence on the estimation of the distance. The other aspect that has to be taken into account is the time response of the sensor because the distance is estimated based on this value.

The infrared (IR) and ultrasonic (US) sensors are the most extensively used in robotics due to their low cost and fast time response, which make them an attractive option for real-time object detection [8]. These sensors use different technologies to estimate the distance to an object. On the one hand, IR sensors use light of wavelength in the range of 760 nm (IR spectrum) for sensing the reflected light intensity. On the other hand, US sensors use a high-frequency sound wave (40 kHz) in order to detect the returning pulses (echo) [9]. IR sensors are used to measure short distances (around 0.1 to 1.5 m) with a narrow (line) beam shape, while US sensors are used to measure long distances (around 0.15 to 6.5 m) with a conical beam shape. The width of the beam is a function of the surface area, frequency, and type of transducers [9,10].

In both cases, the distance estimation depends greatly on what the object is made of. For example, since the IR sensors are a vision-based measurement, the colour of the object and the environment light condition could affect their outcome [11,12]. On the other hand, because of the US sensors are sound-based, they are useful for transparent objects and able to work under poor lighting conditions, but this kind of sensors is much more sensitive to mirror-like surfaces, and the measurement can be affected by environmental parameters such as temperature, humidity, ambient noise, among others [8]. A more detailed description of this issue can be found in [9], where the authors make an analysis of the influence on the behaviour of both sensors for different types of materials such as: cardboard, paper, sponge, wood, plastic, rubber and tile.

Despite the popularity of proximity sensors, they need to be carefully calibrated before using since the measured distance between the robot and the obstacle is relative. A deficient calibration can produce significant errors in the distance estimation, and therefore, the performance of the obstacle avoidance algorithm is poor. If the sensors are included with the mobile robot, the manufacturer usually gives a model to convert raw data into distance, however such model provides an average performance of the distance estimation since this built-in model is not tuned for each sensor. Thus, in order to increase the accuracy of the distance estimation, the traditional method of calibration for a suitable distance model, turns frequently into a manual and time-consuming process [13].

Fusion of different kind of sensors is a fast developing area of research that shows many examples in the literature through the integration of multiple sensors [14,15]. Thus, the integration of the information supplied by US and IR sensors can be a natural extension of previous works in order to provide reliable distance measurements for robot navigation by using these proximity sensors, because the advantages of one sensor compensate for the disadvantages of the other. The fusion of sensors can be implemented by using fuzzy logic [16], neural networks [17,18,19], Kalman filtering [20], or support vector machines among other solutions [14]. Although we can find examples of sensor fusion for robot navigation in the literature, many of the them show only theoretical results [20,21] or quite ad hoc experiments [16,17,18], or they mix the distance model with the collision avoidance algorithm [17,19,22], implying that work on scientific experimental validation of sensor fusion methods for robot navigation still needs further research.

This paper presents a novel approach for modelling the distance to obstacles using the data provided by two types of proximity sensors. The proposed approach is applied on the fusion of IR and US sensor of the very popular Khepera IV robot [23]. Our approach also provides an automatic way to obtain and calibrate the model for distance estimation to obstacles by using artificial neural networks. After obtaining an accurate distance to the objects it is possible to use any kind of collision avoidance algorithm to perform a successful navigation of the robot in a dynamic environment. In order to validate the proposed approach, the results section of the paper provides real experiments of obstacle avoidance with a comparison between the traditional calibration method (provided by the manufacturer) versus the developed approach by using the same collision avoidance algorithm.

The remainder of the paper is organized as follows: Section 2 describes the traditional sensors calibration method and its drawbacks; Section 3 presents the novel method of automatic smart calibration of the sensors; Section 4 presents some results and experiments obtained with the developed method in comparison with the traditional calibration method; and finally, Section 5 shows the main conclusions and future work.

## 2. Traditional Calibration of Proximity Sensors

The traditional calibration method of proximity sensors is a manual repetitive process. It consists in placing some objects around the robot and measuring the raw values given by the sensors (usually voltages). With this values, a model of the sensors is built using a conventional identification method. The result of this procedure are the coefficients of the model (linear or nonlinear) that calculates the distances to the objects using the voltage of the sensors as inputs. Usually the same model is used for all the sensors of the same type, but in practice, not all the sensors work identically and this can introduce erroneous values.

**Figure 1:**
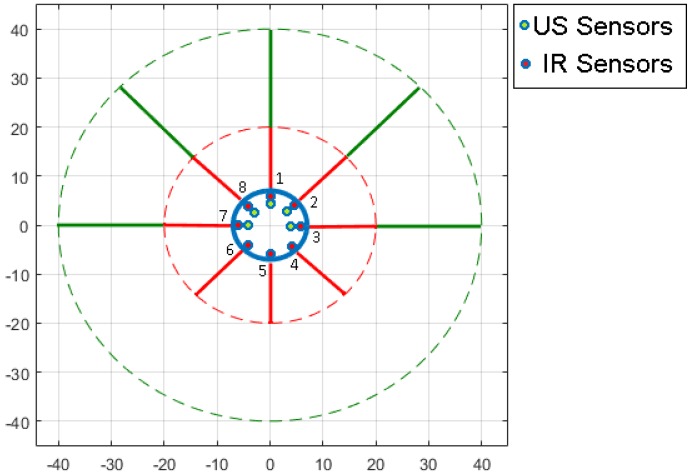
Khepera IV robot sensors distribution.

To carry out the traditional calibration process in the laboratory, two Khepera IV robots [23] are used. Khepera IV is a mobile robot that has been designed for indoor pedagogical purposes and brings numerous features, for example, a colour camera, WI-FI and Bluetooth communications, an array of 8 infrared sensors (IR) for obstacle detection (2 mm to 20 cm) and 5 ultrasonic (US) sensors for long range object detection (20 cm to 40 cm), etc. Figure 1 represents the distribution and the ranges of the proximity sensors in the Khepera IV robot.

The IR sensors (red colour) are distributed every 45${}^{{}^{\circ}}$ around the robot (form 1 to 8). While the US sensors (green colour) are distributed every 45${}^{{}^{\circ}}$ in the front half of the robot (1, 2, 3, 7 and 8).

**Figure 2:**
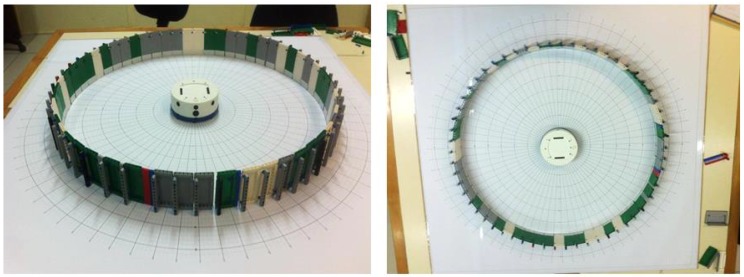
Setup for the traditional calibration method.

Figure 2 shows the set up configuration in the laboratory to carry out the calibration. To start the process, the robot is placed at the center of a “calibration carpet”. Plastic obstacles of different colours conforming a wall concentric to the robot sensors are used to measure their values.

**Figure 3:**
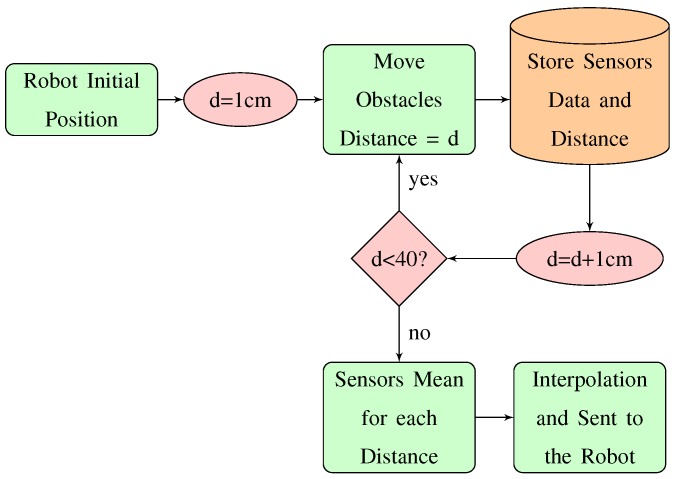
Flow diagram of the traditional calibration procedure.

The robot stores 20 values per sensor and then the obstacles are displaced 1 cm over its radius. The process is repeated from 1 cm to 40 cm for all the sensors. If the distance is less than 40 cm the process is repeated. Otherwise, the data stored in the robot is sent to the PC to calculate the mean value for each sensor/distance. Then, the values are linearly interpolated. Figure 3 shows the flow diagram with the steps to carry out this process. The blocks represented in green colour are related to the robot. The blocks represented in orange colour are related to the PC. And, the pink colour blocks are related to the manually action taken by the user.

**Figure 4:**
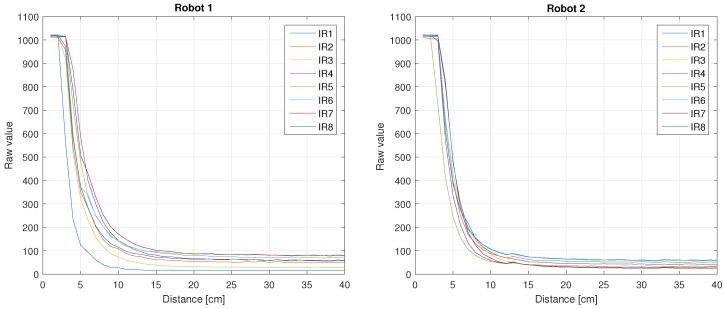
Robots 1 and 2 IR sensors values vs. distance.

Figure 4 shows the results of the previous process for the IR sensors: on the left side, the raw values from the sensors for the Robot 1; and on the right side, the raw values of the Robot 2. As can be seen, the sensors have a nonlinear behaviour for distance values less than 20 cm and the values are different for same sensor/distance (note that all these sensors are of the same type). For distance values greater than 20 cm the behaviour is almost the same. That is why it is important to uses the IR sensors only to measure distances less than 20 cm.

**Figure 5:**
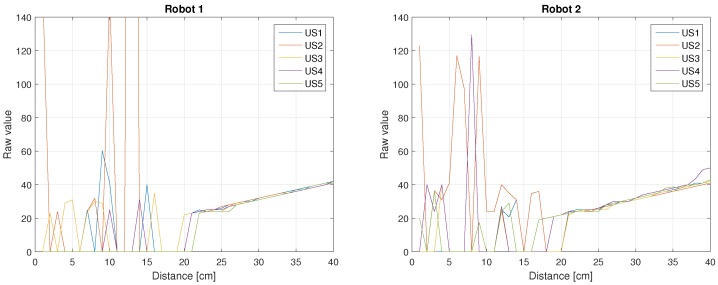
Robots 1 and 2 US sensors.

Figure 5 shows the results of the previous process for the 5 US sensors of Robots 1 and 2. As can be seen, in both cases for distance values less than 20 cm the results are not good. But, for values greater than 20 cm, the results are almost linear. Note that like in the previous case, the values from the sensors in both robots are different, despite the fact that they are the same type of sensor.

Following the traditional method the next step is to calculate the model of both type of sensors with the obtained values. Note that the model for each kind of sensor is different. You can obtain a model that combine these two models into one, like in [24].

In the resulting model, for values less than 20 cm the model of the IR sensors is used and for values grater than 20 cm the US model is used. Note that if you need more accuracy, you can obtain a different model for each combination of US and IR on each robot. This is a heavy work because you need to do this process manually in a repetitive way for each robot/sensor if you want to obtain accurate models for all sensors.

As was mentioned before, the results of the manual calibration shows that the information of the sensor must be fused to detect obstacles in the entire range (from 0 cm to 40 cm) in front half of the robot. While in the half back of the robot the obstacles can only be detected from 0 cm to 20 cm.

**Figure 6:**
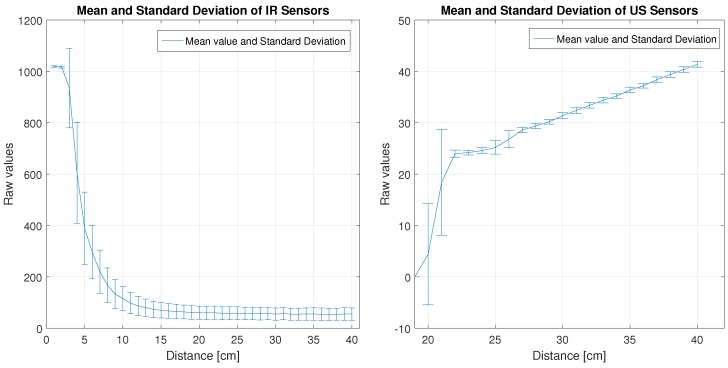
Mean and Standard Deviation of the IR and US sensors of Robot 1.

Figure 6 shows the mean value and the standard deviation of the sensors measurements for the Robot 1. On the left side are represented these values for the IR sensors. As can be seen, for short distances (between 2 cm and 15 cm) there are appreciable differences between the raw values of the sensors for the same distance. Which means that the use of the same model for each sensor may cause erroneous measurements in this range. For distances greater than 20 cm the differences are smaller.

On the right side of Figure 6, the values of the US sensors are shown. As can be seen, for distances between 20 cm and 26 cm there are appreciable differences between the raw values of the sensors for the same distance. For distances greater than 27 cm the differences are smaller.

Next section presents the main contribution of this work: An automatic smart method to carry out the calibration of the sensors based on data and using an artificial neural network.

## 3. Automatic Smart Calibration Method

As was explained before, the traditional calibration is a manual and repetitive process that can be heavy depending of the accuracy that you need to obtain from the model of your sensors. In this section the novel automatic method based on data and using artificial neural networks is proposed.

### 3.1. Platform Used in the Laboratory

**Figure 7:**
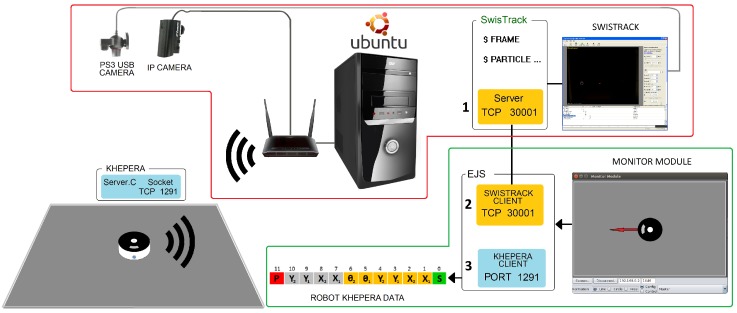
Hardware and software components of the platform.

The method needs to obtain the absolute position of the robot each step execution. This task is carried out by the platform presented in [25], which is a previous work of the authors. This platform implements an IPS (Indoor Positioning System) to locate the robot. Figure 7 shows the architecture of the platform whose components are the following: (a) A personal computer (PC) with the Ubuntu Linux operating system that executes the software tools (Swistrack [26,27] and Monitor Module); (b) a PlayStation 3 (PS3) USB camera (fixed to the ceiling) connected via USB to the PC (The images of this camera are processed by *Swistrack* to obtain the position of the robot); (c) a Wi-Fi Router that communicates the Khepera IV robot with the PC; (d) an IP camera to show the performance of the experiments.

### 3.2. Automatic Smart Calibration Method

The method consists of constructing the model of the proximity sensors automatically. For this, some obstacles are placed in known locations in the workspace. Then the robot navigates through the workspace storing the values from the proximity sensors. With the known positions of the obstacles and the information provided by the sensors of the robot, a neural network is built offline based on this data. The resulting model gets the raw values from the sensors as input and provides as output the distance to the obstacles around the robot. Note that this method needs to know the absolute position of the robot during the experiment.

**Figure 8:**
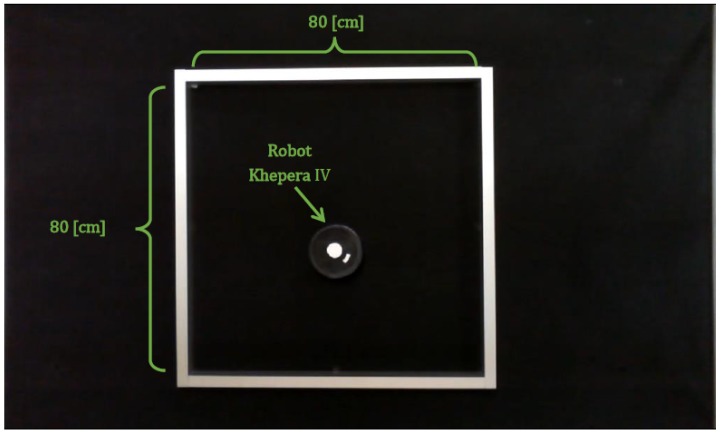
Set up of the workspace.

**Figure 9:**
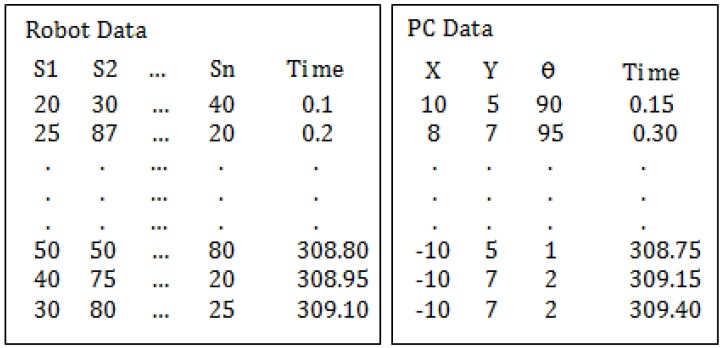
Data stored in the robot and in the PC.

**Figure 10:**
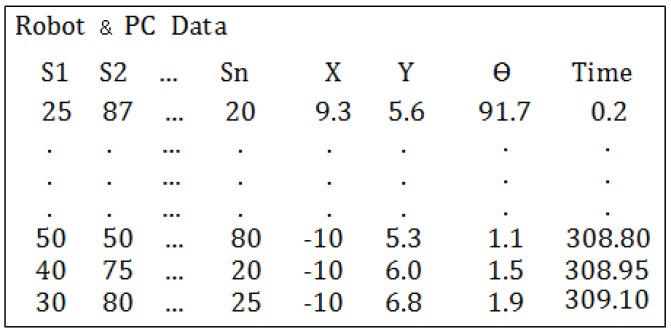
Robot and PC data merged.

**Figure 11:**
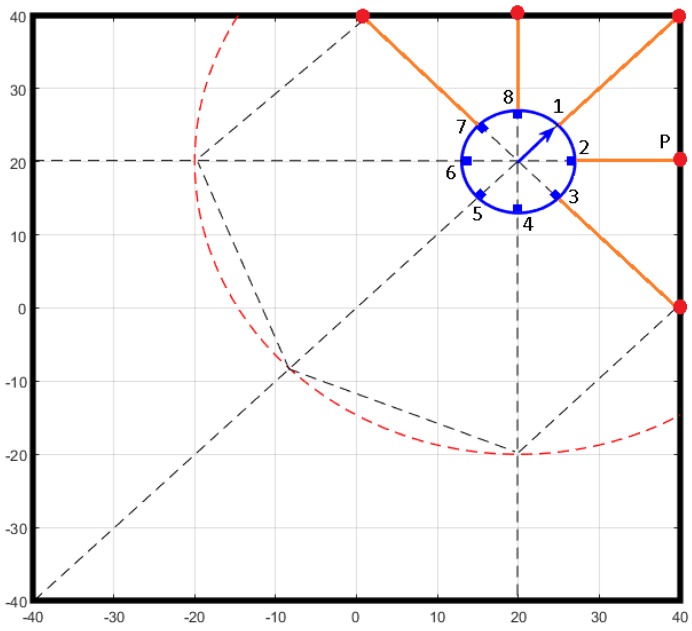
Distance from the sensors to known obstacles (walls).

**Figure 12:**
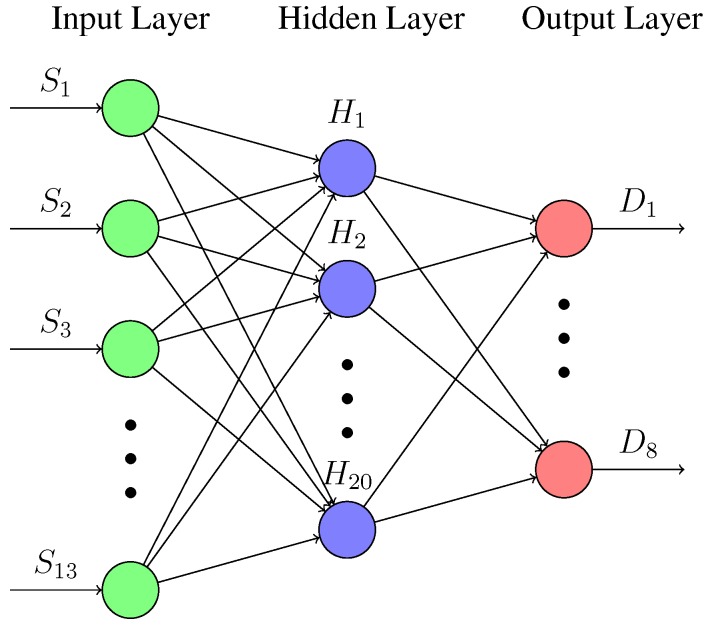
Artificial neural network implemented.

**Figure 13:**
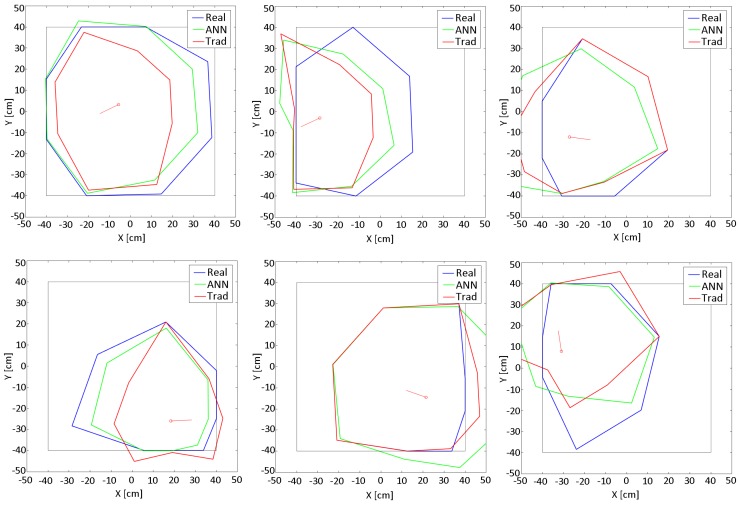
Images sequence of a test in the workspace.

The method consist of the following steps:

1. Set up of the workspace: A square shaped wall (80 cm) is placed in the Arena. The robot is placed in the center of the square with a known orientation. Note the model will work until 40 cm. The robot starts to move implementing the Braitenberg algorithm [28] to avoid the obstacles. Figure 8 shows the aerial view of the set up.
2. Data acquisition: The robot stores the data of the sensors (S${}_{1}$, S${}_{2}$, ..., S${}_{n}$) and the time during its movement. On the other hand, the PC stores the position of the robot (coordinates and orientation) and also the time during the experiment. At the beginning of the process the time in the PC and the robot starts in 0. But the sample time is different for both because they run independently. Which means that the acquired data must be synchronized in time by interpolation.
  After the experiment finishes, the data of the robot is copied to the PC. Since the time stored in the robot and in the PC are not coincident this data needs to be adjusted and synchronized. This process is carried out of a code developed in MATLAB to do this task. Figure 9 shows an example of the data stored in the robot and in the PC.
3. Data conditioning and synchronization: The data is adjusted by liner interpolation of the time, using Equation (1):
  (1) $$\left\{\begin{array}{cc} X\left(t\right)=\left(\frac{X\left(T_{1}\right)-X\left(T_{0}\right)}{T_{1}-T_{0}}\right)*\left(t-T_{0}\right)+X\left(T_{0}\right)\\ Y\left(t\right)=\left(\frac{Y\left(T_{1}\right)-Y\left(T_{0}\right)}{T_{1}-T_{0}}\right)*\left(t-T_{0}\right)+Y\left(T_{0}\right), & T_{0}<t\leq T_{1}\\ \theta \left(t\right)=\left(\frac{\theta \left(T_{1}\right)-\theta \left(T_{0}\right)}{T_{1}-T_{0}}\right)*\left(t-T_{0}\right)+\theta \left(T_{0}\right) \end{array}\right.$$
  where $T_{0}$ and $T_{1}$ are the initial and final time of the position data where the interpolation is made. *t* is the time that is evaluated which corresponds with the data of the sensors. $X\left(t\right)$, $Y\left(t\right)$ and $\theta \left(t\right)$ are the pose of the robot in time *t*. Figure 10 shows the results of this process, where the data of the robot sensors and the robot positions are merged.
  Once the position of the robot is obtained, it is necessary to obtain the positions of all sensors for each robot position. The positions of the sensors only depend on the pose of the robot. Using Equation (2) this position can be calculated.
  (2) $$\left\{\begin{array}{c} X_{sn}=radius_{robot}*\cos\left(angle_{sn}+\theta_{robot}\right)+X_{robot}\\ Y_{sn}=radius_{robot}*\sin\left(angle_{sn}+\theta_{robot}\right)+Y_{robot} \end{array}\right.$$
  Figure 11 represents the distances from the sensors to the walls (yellow colour). The black solid lines are the walls. The blue circle is the robot, the small rectangles are the sensors and the blue arrow represents the orientation of the robot. The dashed lines represent the detection directions of the senors. The dashed circle (40 cm of radius) represents the maximum detection range of the sensors.
  The distance from each sensor to the walls is calculated by the intersection points (red) between the detection direction lines and the walls. Equation (3) shows how the coordinates of the intersection point P($X_{m}$;$Y_{m}$) are calculated for sensors 1, 2 and 3.
  (3) $$\left\{\begin{array}{c} X_{m}=40\\ Y_{m}=\left(\frac{Y_{robot}-Y_{sn}}{X_{robot}-X_{sn}}\right)*\left(X_{m}-X_{sn}\right)+Y_{sn} \end{array}\right.$$
  For sensors 7 and 8 is the same process only changing the axis because in this case the intersection is with the wall x = 40. Having these intersection points the distances and the angles are easily calculated by Equation (4). Finally the obtained data is filtered.
  (4) $$\left\{\begin{array}{c} \theta_{m}=atan\left(\frac{Y_{robot}-Y_{m}}{X_{robot}-X_{m}}\right)\\ d_{m}=\sqrt{{\left(X_{sn}-X_{m}\right)}^{2}+{\left(Y_{sn}-Y_{m}\right)}^{2}} \end{array}\right.$$
4. Artificial Neural Network (ANN): With the calculated values and the raw data of the sensors, an ANN is built using the Neural Network Toolbox 9.0 of MATLAB [29], to obtain the model. Figure 12 shows a representation of the ANN implemented based on Levenberg-Marquardt method [30].
  The input layer receives the 13 raw values (S${}_{1}$–S${}_{13}$) of the sensors (8 IR + 5 US). While the hidden layer has 20 neurons (H${}_{1}$–H${}_{20}$) with the Hyperbolic Tangent as activation function. The output layer has 8 neurons with the Identity as activation function. The outputs (D${}_{1}$–D${}_{8}$) are distances to the obstacles in the positions of the sensors (every 45${}^{{}^{\circ}}$ around the robot). The training was carried out with the 70% of the acquired data, the validation with another 15% and the testing with the remaining 15%. Note that the parameters of the ANN (number of neurons in the hidden layer and activation functions) have been determined empirically by trial and error after multiple tests.
5. Testing the resulting model: Figure 13 shows a test developed in the workspace where the model was obtained. The sequence of images shows the displacement of the robot through the scenario. The black lines are the walls of the square (80 cm by 80 cm). The red small line represents the position and orientation of the robot. The performance of the models is drawn around the robot: (a) Blue lines: are the real distances to the obstacles (less than 40 cm and calculated with the intersection process described before); (b) Green lines: are the results of the the ANN model, taking and fusing the raw inputs from the sensors and providing the 8 distances; and (c) Red lines: are the distances to the obstacles with the traditional calibration method.
  As can be seen, all the models have different behaviors for different situations. The better results are obtained in general for distances less than 30 cm. But there are appreciable differences between the results of the half front part of the robot and the back half. This is due to the fact that the back part of the robots does not have US sensors. Even so, these results are more than acceptable compared with the majority of existing obstacle avoidance methods, and based on the maximum velocity of this robot and its steering characteristics.
  Table 1 shows the Mean Absolute Percentage Error (MAPE) of the previous tests with respect to the real distances. The rows represent the distances (D${}_{1}$–D${}_{8}$) to the obstacles from the position of the sensors. The columns represent the results for both models (Artificial Neural Network (ANN) and Traditional (Trad)) for Robots 1 and 2.

In all the cases, the smallest error values are represented in bold. As can be seen, the results for both robots are different. In the case of Robot 2 the traditional method shows worse results than the ANN method. In the case of Robot 1, the behavior is similar to the previous one. Only distances 1 and 8 present worse results. In general, for both cases the ANN model presents better results than the traditional model.

**Figure 14:**
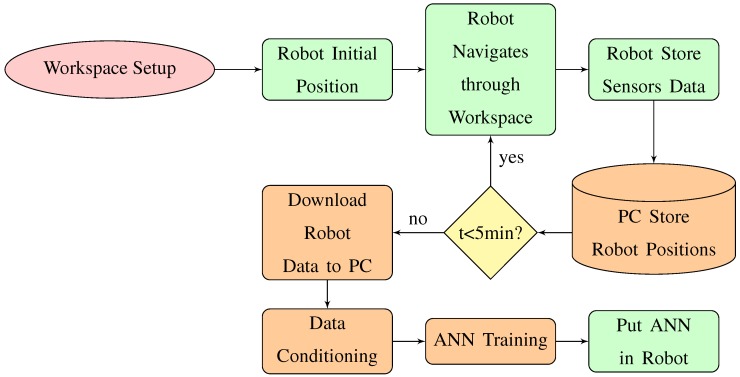
Flow diagram of the developed calibration procedure.

Figure 14 shows the flow diagram of the developed method. The block related to the robot are represented in green colour. The orange colour represents the blocks related to the PC. Note that the ANN model is obtained offline with the acquired data by the robot and by the PC. After that the model is implemented in the robot. The model gets the raw values from the sensors and gives the distances to the objects in the 8 directions where the sensors are located in the robot.

Note that this method has been developed for the Khepera IV robot. The properties of the robot and platform have a direct influence in the performance of the method. But it does not mean that the method cannot be implemented in other robots/platforms that use proximity sensors to estimate the distance to obstacles. To do that, one important element to be taken into account is the use of an indoor positioning system which gives the absolute position of the robot in running time. Other important aspect is the location of the sensors around the robot. This configuration has to be considered when the distances from each sensor to the walls (see Figure 11) are calculated by using Equations (2)–(4).

## 4. Experiment of Position Control with Obstacles Avoidance

**Figure 15:**
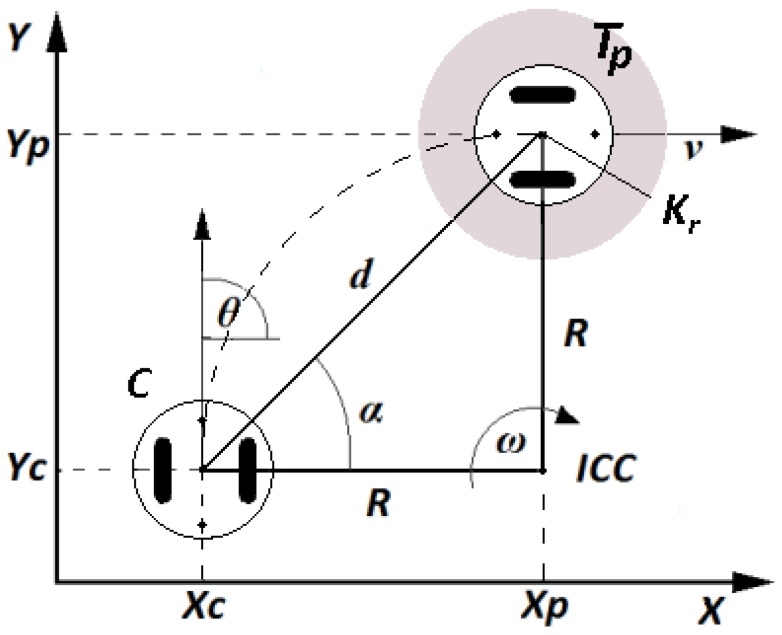
Position control problem.

This experiment is titled position control or point stabilization of a differential wheeled mobile robot. This problem has been widely studied mainly from an automatic control perspective [31]. The objective of the experiment is to drive the robot from the current position $C(x_{c},y_{c})$ and orientation (θ) to the target point $T_{p}\left(x_{p},y_{p}\right)$. Figure 15 shows a representation of the variables involved in this experiment.

**Figure 16:**
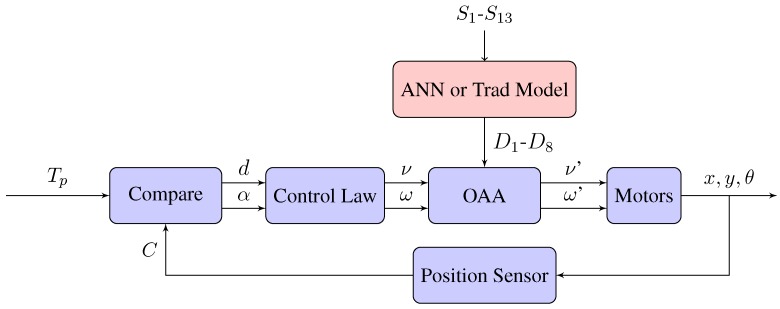
Block diagram of the position control problem.

Figure 16 shows the feedback control loop block diagram of this experiment but in this case, including the obstacle avoidance algorithm and the model obtained with the developed method.

The block Compare calculates the distance (*d*) and the angle α to the target point ($T_{p}$) from the current position of the robot (*C*). The block Control Law tries to minimize the orientation error, $\theta_{e}=\alpha -\theta$, and at the same time, to reduce the distance to the target point (d=0) by manipulating the control signals (linear velocity (ν) and angular velocity (ω) of the robot). Equations (5) and (6) show the implementation of this control law based on [32].

(5) $$\nu =\left\{\begin{array}{cc} \nu_{max} & \mathrm{if}\left|d\right|>K_{r}\\ d\left(\frac{\nu_{max}}{K_{r}}\right) & \mathrm{if}\left|d\right|\leq K_{r} \end{array}\right.$$

(6) $$\omega =\omega_{max}sin\left(\theta_{e}\right)$$

The block OAA represents the Obstacle Avoidance Algorithm, in this case: Braitenberg algorithm [28], in which the robot’s sensors are tied directly to the motor controls and motor speeds respond to the sensor input directly. The Model block represents the developed algorithm which obtains the distances to the obstacles (D${}_{1}$–D${}_{8}$) from the raw data of proximity sensors (S${}_{1}$–S${}_{13}$). If no obstacle is detected, the output velocities of the block OAA are the same as its inputs (ν, ω).

The Braitenberg algorithm [28] creates a weighted matrix that converts the sensor inputs into motor speeds. This matrix is a two-dimensional array with the number of columns corresponding to the number of obstacle sensors (8) and the number of rows corresponding to the number of motors (2). The weights of the matrix are determined empirically depending on the location of the sensors in the robot.The 8 sensors of the Khepera IV robot are numbered clockwise beginning with the front sensor. Equation (7) represents the mentioned matrix where, for example, the term $W_{LS_{1}}$ represents the weight of the sensor $S_{1}$ in the speed of the left motor. Equation (8) represents the raw data of the proximity sensors at each time.

(7) $$W=\left(\begin{array}{cccccc} W_{LS_{1}} & W_{LS_{2}} & . & . & . & W_{LS_{8}}\\ W_{RS_{1}} & W_{RS_{2}} & . & . & . & W_{RS_{8}} \end{array}\right)$$

(8) $$S={\left(\begin{array}{cccccc} S_{1} & S_{2} & . & . & . & S_{8} \end{array}\right)}^{T}$$

With these matrices, the velocities for each motor are calculated as is shown in Equation (9). Where ($S_{max}$) represents the maximum value of the sensor output.

(9) $$\nu_{L,R}=W*\left(1-\left(S/S_{max}\right)\right)$$

**Figure 17:**
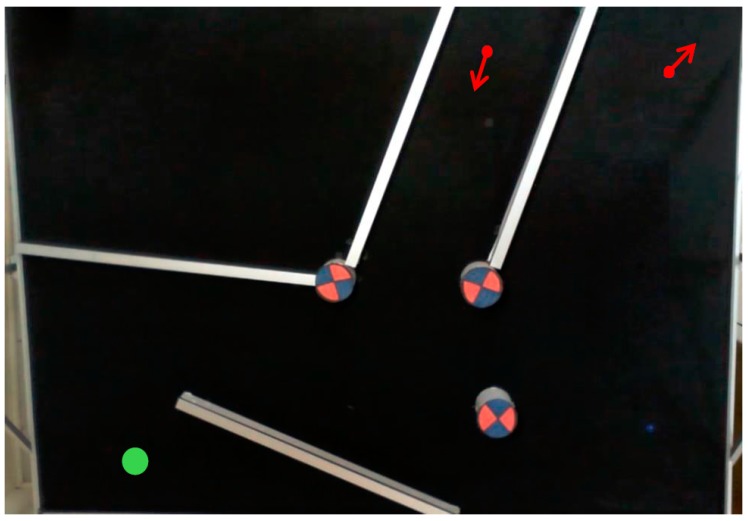
Scenario for obstacle avoidance experiments.

Figure 17 shows the top view of the set-up of the scenario to carry out two experiments where the elements are the following: (a) red arrows represent the starting points and orientation of the robot; (b) green circle represents the target point for both cases; and (c) obstacles are walls (white colour) and cylinders (blue/red colours).

**Figure 18:**
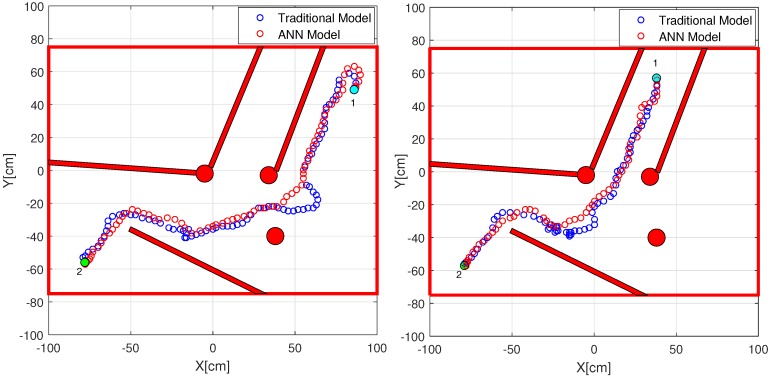
Obstacles avoidance experiment results for Robot 2.

Figure 18 shows the results of both experiments with the ANN and Traditional models. The red lines and circles are the obstacles. In both cases, the robot begins the experiment starting at point 1 (cyan circle) and it must reach the target point 2 (green circle). The small blue points represent the position of the robot during the experiment for the traditional calibration model (Trad), while the small red points represent the position of the robot for the developed method (ANN).

In both cases the robot reaches the target point avoiding the obstacles that it finds on its way. As can be seen, the behavior of the robot with the ANN model has better performance than with the traditional model. These differences show that you can use the functions provided by the manufacturer or calibrate the sensors manually using the traditional method. In both cases you need to take into account that your obstacle avoidance algorithm can be working correctly for the values of distances that it is receiving. However, the real problem is that the sensors can be providing erroneous values due to bad calibration or the use of a poorly tuned model for all the sensors.

Table 2 shows the Mean Absolute Percentage Error (MAPE %) of these experiments presented in Figure 18. The columns represent the following: (Exp) the number of the experiment, (Method) the model, (MAPE) the mean values of the distance errors (from $D_{1}$ to $D_{8}$) and (Time) the time to reach the target point.

The results represented in bold indicate the smallest values of the errors and time. As can be seen, for all experiments the ANN model presents less mean error than the traditional model. In addition, in both experiments the robot reaches the target point in less time, which means that the robot describes a smoother and more direct path to the target point due to better detection and avoidance of the obstacles.

**Figure 19:**
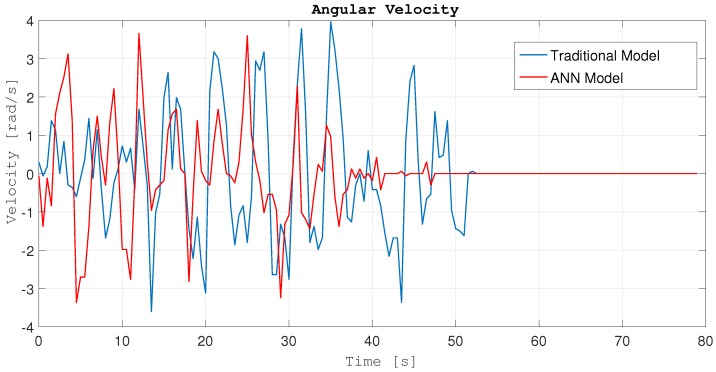
Angular velocities.

Additionally, Figure 19 shows the angular velocity of the robot in the second experiment (right side of Figure 17) for both models: blue line represents the traditional model; and red line represents the ANN model.

**Figure 20:**
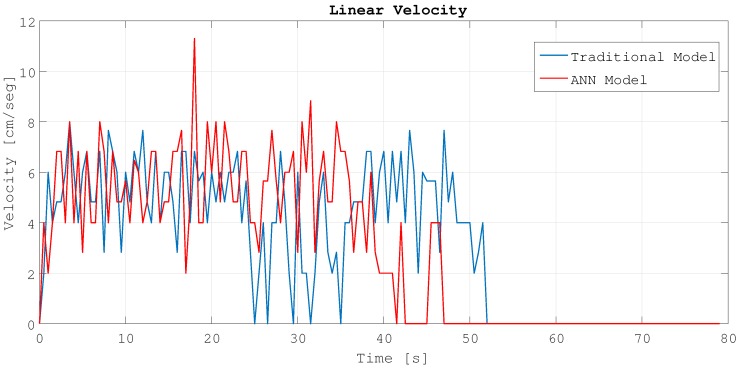
Linear velocities.

As can be seen, for the same experiment, with the ANN model the angular velocity has less oscillations than with the traditional model. Due to better detection and avoidance of the obstacles, the behavior of the robot is smoother because it can avoid the obstacles in a better way with less direction changes. Because it reaches the target point first, that means that the trajectory is more efficient. Figure 20 shows the linear velocities for the same experiments and models.

In the case of the linear velocities, also the ANN model has less oscillations and it becomes zero first. This means that the robot reaches the target point faster than with the traditional model and with a more constant velocity.

## 5. Conclusions

The most common proximity sensors in mobile robotics are the US and IR sensors. They are widely used for obstacle detection without physical contact. But they introduce uncertainties in the measurements due to their mode of operation: they need a feedback signal that depends on the surface of the object and the ambient conditions. Another important issue for these sensors is the calibration of their outcomes. Usually one has to obtain a model that converts the raw measurements provided by the sensors in distances. The method for building this model is a repetitive and lengthy process that usually is done manually. Moreover, the obtained model cannot provide the same distance even for two sensors of the same type (due to the uncertainties introduced by the surfaces of the objects). That is why in some situations it could be useful to gather all measurements provided by different sensors and combine them in order to get an accurate model to estimate the distances to the obstacles around the robot.

This paper presents a novel approach to get an obstacle detection model based on the combination of sensor data. The method is automatic and based on data using machine learning. The method implements an artificial neural network to obtain the model of the sensors. The model receives the raw values from the 13 sensors (8 IR + 5 US) and provides the distances to the obstacles around the robot (8 values). This means that the method combines the information from both kinds of sensors and builds a unique model for obstacle distances.

The developed method has been designed for the Khepera IV robot in an indoor environment with an IPS to obtain its absolute position in running time. Although it has been designed for these characteristics, this method can be implemented to different platforms. The fundamental points that have to be taken into account are the indoor positioning system and the location of the sensors around the robot.

To show the performance of the developed model, some results are provided and discussed. The experiment of position control with obstacles avoidance based on the Braitenberg algorithm was selected. To test it, two different scenarios were used for this experiment. In addition, the velocity of the robots was analyzed in both cases. The comparison between the traditional and the developed method shows the improvement in the results.

This method is undoubtedly an advance in the calibration of proximity sensors because it eliminates the traditional laborious method and provides better results. Future works are related to the implementation of this method in other platforms with other kinds of robots and sensors.

## Figures and Tables

**Table 1:** Mean Absolute Percentage Error for both models and robots.

MAPE (%)				
	Robot 1		Robot 2	
	ANN	Trad	ANN	Trad
D${}_{1}$	55.77	**54.33**	**30.45**	38.49
D${}_{2}$	**67.06**	79.01	**25.56**	40.09
D${}_{3}$	**66.64**	76.20	**21.05**	29.24
D${}_{4}$	**50.71**	66.46	**12.68**	23.44
D${}_{5}$	**44.35**	51.74	**9.50**	17.17
D${}_{6}$	**47.61**	51.12	**11.76**	21.54
D${}_{7}$	**46.29**	56.80	**21.68**	39.24
D${}_{8}$	58.09	**55.37**	**32.03**	42.45

**Table 2:** Results of three experiments for both models.

Exp	Method	MAPE (%)	Time (s)
1	Trad	83.9	59.4
1	ANN	**65.5**	**50.8**
2	Trad	88.8	60.4
2	ANN	**58.4**	**51.3**
